# Etiology, Clinical Features, and Outcomes of Acute Poisoning Among Children at a Provincial Pediatric Hospital in Vietnam: Retrospective Descriptive Study

**DOI:** 10.2196/97091

**Published:** 2026-10-09

**Authors:** Cao Thanh Vu, Tien Dat Pham, Anh Tuyet Bui, Huy Quan Dam, Van Hung Lanh, Pak Chhengpay, Thi Hoa Luu, Thi Thuyet Luong, Thi Thu Huyen Pham, Minh Manh To, Van Lap Khuc

**Affiliations:** 1 Thai Binh University of Medicine and Pharmacy, 373 Ly Bon, Hung Yen, Hưng Yên Province, Vietnam, 84 2273838545; 2 Thai Binh Pediatric Hospital, Hung Yen, Hung Yen, Vietnam

**Keywords:** pediatric poisoning, acute poisoning, children, accidental poisoning, toxic exposure, clinical outcomes

## Abstract

**Background:**

Acute poisoning in children remains a significant cause of emergency admission and is largely preventable. Its epidemiological profile varies across settings according to age, household environment, and locally available toxic agents. Understanding the patterns, management, and outcomes of pediatric poisoning is important for improving prevention and clinical care.

**Objective:**

This study aimed to characterize the demographic and exposure patterns, treatment practices, and in-hospital outcomes of acute poisoning among children treated at a provincial pediatric hospital in Vietnam.

**Methods:**

We conducted a hospital-based retrospective descriptive study of children aged <16 years treated for acute poisoning at Thai Binh Pediatric Hospital, Vietnam, from January 1, 2023, to December 31, 2025. Hospital records were screened using prespecified eligibility criteria. Data included age, sex, time from poisoning to hospital presentation, exposure location and intent, route and agent of poisoning, recorded treatment measures, and in-hospital outcomes. Analyses were descriptive, with variable-specific denominators used when data were missing.

**Results:**

Of the 326 records identified, 276 were included in the final analysis. Children aged 1 to 5 years accounted for the largest proportion (n=151, 54.7%), and 53.6% (n=148) were male. Time from poisoning to hospital presentation was not reported for 12.3% (n=34) of the children; 67.4% (n=186) presented to the hospital within 6 hours after poisoning. Poisoning occurred mainly at home (n=266, 96.4%) and was predominantly unintentional (n=265, 96%). All 11 intentional poisonings occurred in children aged >10 years. Ingestion was the main route of exposure (n=220, 79.7% documented exposure routes). Chemical substances, medications, and food were prominent agent groups among the classifiable agents reported in the dataset. Intravenous fluids were recorded in 80.4% (n=222) of children, gastric lavage in 26.1% (n=72), and single-dose activated charcoal in 24.3% (n=67). Overall, 97.5% (n=269) of children were discharged from the study hospital and 2.5% (n=7) were transferred to a national hospital. Final outcomes after transfer were unavailable. No deaths occurred at the study hospital.

**Conclusions:**

Pediatric acute poisoning treated at this provincial hospital mainly affected young children and most often resulted from unintentional exposure in the home. The study identifies locally relevant exposure patterns that can guide household prevention and age-specific risk reduction. Most children were discharged from the study hospital, but final outcomes were unavailable for transferred patients; therefore, the findings should not be interpreted as demonstrating uniformly favorable outcomes or the appropriateness of specific treatments.

## Introduction

Acute poisoning in children is defined as the occurrence of harmful clinical effects following exposure to a toxic substance over a short period, usually through ingestion, inhalation, dermal absorption, or other acute routes of contact. It remains a major pediatric emergency and a significant public health concern because it can result in emergency department visits, hospitalization, intensive care admission, long-term complications, and death when recognition or treatment is delayed [1-3]. The burden of acute poisoning is especially important in children because of their developmental characteristics and behavioral vulnerability. Young children frequently explore their environment by putting liquids, tablets, plants, and household products into their mouths, whereas older children and adolescents are more likely to experience poisoning related to intentional self-harm, substance misuse, or unsafe experimentation [1,4,5]. In addition to its direct clinical impact, acute poisoning imposes considerable emotional and economic burdens on families and increases pressure on health care systems, particularly in low- and middle-income countries where access to poison control services and specialized emergency care may be limited [6].

The epidemiology of childhood acute poisoning varies across countries and regions, but several consistent patterns have been reported. In many studies, children aged <5 years account for the largest proportion of poisoning cases because of their natural curiosity and high risk of accidental ingestion [7,8]. In contrast, intentional poisoning is more frequently observed among adolescents and is often associated with psychosocial distress and easier access to medicines or toxic chemicals [4,5,9]. The epidemiological profile of poisoning also differs according to setting. In some regions, pharmaceutical agents are the leading cause, whereas in rural and agricultural areas, pesticides, rodenticides, hydrocarbons, and household chemicals are more prominent [10-13]. Although many children with acute poisoning recover with timely supportive care, severe cases may lead to respiratory failure, cardiovascular instability, neurological impairment, metabolic disturbances, prolonged hospitalization, or death [13-15].

The causes of acute poisoning in children are diverse and strongly influenced by local context. Common toxic agents include medications, household cleaning products, pesticides, corrosives, hydrocarbons, cosmetics, plants, and industrial chemicals [1,3,7]. Household practices such as storing chemicals in drink bottles or keeping toxic substances within children’s reach may increase the risk of accidental poisoning. In Vietnam, local exposure patterns may vary according to household practices, availability of medicines and chemicals, agricultural activities, and access to emergency care [16].

Despite the growing literature on pediatric poisoning, recent evidence from Vietnam remains limited. A 10-year study from Haiphong Children’s Hospital reported that children aged 1 to 5 years were most frequently affected. Most exposures were unintentional, and nonpharmaceutical chemicals and medications were the leading agent groups [16]. Additional provincial data can determine whether these patterns persist in other settings and can identify locally prominent agents relevant to prevention and service planning. Therefore, this study aimed to characterize the demographic and exposure patterns, treatment practices, and in-hospital outcomes of acute poisoning among children treated at Thai Binh Pediatric Hospital.

## Methods

### Study Design and Setting

This study was a hospital-based retrospective study conducted at the Emergency and Poison Control Department of Thai Binh Pediatric Hospital, a provincial grade 1 pediatric specialty hospital in Thai Binh province. In July 2025, Thai Binh and Hung Yen provinces were merged into a single province named Hung Yen. Thai Binh Pediatric Hospital provides pediatric care to children from Thai Binh and neighboring areas. It also supports lower-level health facilities in pediatric care. The Emergency and Poison Control Department is a frontline 24-hour unit that receives and manages pediatric emergencies, including acute poisoning. It has facilities for monitoring, resuscitation, gastrointestinal decontamination, and selected antidotal treatment.

### Study Population

The study population included all children aged <16 years with a diagnosis of acute poisoning and treated at Thai Binh Pediatric Hospital from January 1, 2023, to December 31, 2025. Case identification used the hospital’s electronic medical record system. A total of 326 records with an acute poisoning diagnosis were identified during the study period. After application of the eligibility criteria and exclusion of incomplete records, 276 children were included in the final analysis.

### Inclusion Criteria

Children were eligible for inclusion if they were aged <16 years and had a diagnosis of acute poisoning. Acute poisoning was defined when at least two of the following three criteria were met: (1) documented evidence of toxic exposure, such as a witnessed ingestion or exposure, a history provided by the child or caregiver, physical evidence (eg, medication packages or opened chemical containers), characteristic odors, or evidence of bites or stings; (2) clinical manifestations consistent with acute poisoning, including sudden abnormal symptoms in a previously healthy child that could not be explained by another cause; and (3) toxicological testing showing a toxic substance in biological samples consistent with the clinical presentation. Patients with anaphylactic shock or other allergic reactions after drug use, chronic poisoning, or records considered insufficient for study abstraction were excluded from the study.

### Data Collection

Data were abstracted from medical records using a structured case record form developed for the study. Variables included age, sex, exposure location, time from poisoning to hospital presentation, exposure intent, route of exposure, poisoning agent, recorded treatment measures, and in-hospital outcome. Exposure intent (intentional or unintentional) was abstracted from the treating clinician’s documentation, which incorporated the recorded circumstances of exposure and history obtained from the child and/or caregiver. If the documentation was insufficient to determine intent, the case was to be coded as uncertain; all included cases had a recorded classification. Because this classification depended on retrospective clinical documentation and family or child history, misclassification was possible, particularly when self-harm, neglect, or family circumstances were incompletely documented.

Regarding poisoning agents, food included spoiled or unhygienically prepared food, poisonous mushrooms, and food products or additives of unknown origin. Other medications included medications outside the prespecified medication groups, such as antiepileptic drugs, oral contraceptives, vitamin supplements, and traditional medicines. Other chemical substances included agents outside the prespecified chemical categories, such as mothballs, cajuput oil, 90% ethanol, petroleum jelly, gel ice packs, and mercury. Snakebite referred to snake envenomation, whereas insect stings or bites referred to bee stings. When more than one substance was documented, the agent category reflected the substance judged by the treating team to be responsible for the clinical toxidrome, and 2 such coexposure cases were recorded. The exposure route could include more than one route. Data were entered into Microsoft Excel for management and cleaning before analysis.

### Statistical Analysis

RStudio (Posit PBC) was used for statistical analysis. Only descriptive statistics were applied. Categorical variables were described using frequency and percentage, while continuous variables were summarized as mean and SD or median and IQR, as appropriate. No imputation was performed for missing data because the study did not estimate associations or model-based effects, and the analyses were limited to descriptive summaries. A formal test for missing completely at random was not performed because missing data were handled using transparent, variable-specific denominators rather than inferential modeling. The number of missing observations was also reported for each variable, where applicable.

### Ethical Considerations

This study was conducted using clinical information obtained from eligible pediatric patients treated at Thai Binh Pediatric Hospital during the study period. All collected patient information was kept strictly confidential and was used solely for research purposes. Personal identifiers were not disclosed in the study database, analysis, or reporting. This study protocol was approved by the institutional review board of Thai Binh University of Medicine and Pharmacy (2240.35.C11; issued on November 11, 2025). Approval covered the retrospective review of medical records of children admitted from January 1, 2023, through December 31, 2025. Case identification and research data extraction began in January 2026. No records were accessed or extracted for research purposes before approval. Individual informed consent was waived for this retrospective medical record review. Data were deidentified for analysis, and personal identifiers were not included in the analytic dataset or reports. No participant compensation was provided.

## Results

Of the 326 records identified in the hospital database, 276 were included in the final analysis (Figure 1). The mean age was 5.2 (SD 4.1) years. Children aged 1 to 5 years accounted for 54.7% (n=151), followed by those aged 6 to 10 years at 20.3% (n=56), those aged >10 years at 15.9% (n=44), and infants aged <1 year at 9.1% (n=25). The proportion of male patients was slightly higher than that of female patients, accounting for 53.6% (n=148) and 46.4% (n=128), respectively. In total, 22.1% (n=61) of patients were hospitalized within 1 hour and 45.3% (n=125) were admitted within 1 to 6 hours after poisoning. The time from poisoning admission to hospitalization was not recorded in 12.3% (n=34) of cases (Table 1).

Poisoning occurred mainly at home, accounting for 96.4% (n=266) of the 276 cases. Most events were unintentional (n=265, 96%), while 4% (n=11) of cases were intentional poisonings that occurred in children older than 10 years. Ingestion was the main route of poisoning, accounting for 79.7% (n=220) of documented exposure routes, followed by bites in 17.8% (n=49) of cases (Table 2).

The most frequent poisoning agents were chemical substances (98/276, 35.5%), medications (69/276, 25%), and food (58/276, 21%). Most exposures occurred among children aged 1 to 5 years (Table 3).

**Figure 1.**
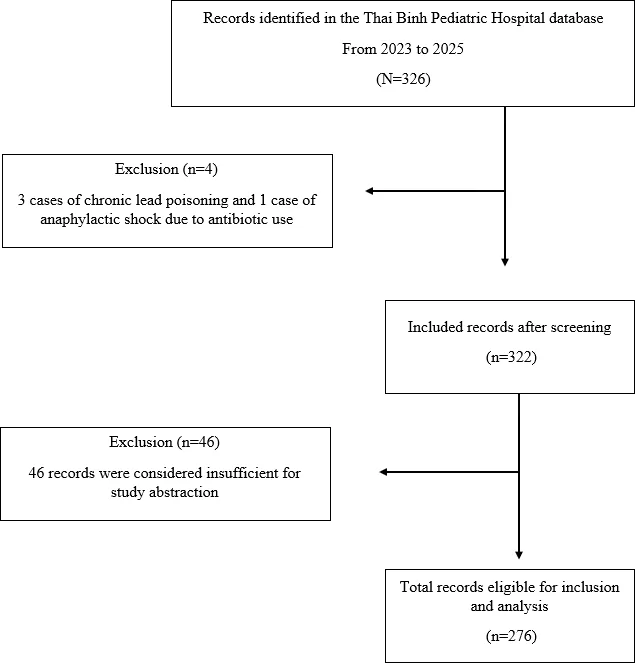
Participant flow diagram.

**Table 1.** Characteristics of the included patients (N=276).

	Patients, n (%)
Age (years)	
<1	25 (9.1)
1‐5	151 (54.7)
6‐10	56 (20.3)
>10	44 (15.9)
Sex	
Female	128 (46.4)
Male	148 (53.6)
Time from poisoning to hospitalization (hours)	
<1	61 (22.1)
1‐6	125 (45.3)
7‐12	25 (9.1)
>12	31 (11.2)
Missing data	34 (12.3)

**Table 2.** Location, mode of poisoning, and route of poisoning.

	<1 year, n (%)	1-5 years, n (%)	6-10 years, n (%)	>10 years, n (%)
Location				
Home (n=266)	24 (9)	149 (56)	54 (20.3)	39 (14.7)
School (n=4)	0 (0)	2 (50)	2 (50)	0 (0)
Others (n=6)	1 (16.7)	0 (0)	0 (0)	5 (83.3)
Mode of poisoning				
Unintentional (n=265)	25 (9.4)	151 (57)	56 (21.1)	33 (12.5)
Intentional (n=11)	0 (0)	0 (0)	0 (0)	11 (100)
Route of poisoning^a^				
Ingestion (n=220)	21 (9.5)	131 (59.5)	36 (16.4)	32 (14.5)
Inhalation (n=4)	2 (50)	1 (25)	0 (0)	1 (25)
Skin or eyes (n=6)	1 (16.7)	4 (66.6)	1 (16.7)	0 (0)
Bites (n=49)	1 (2)	18 (36.7)	19 (38.8)	11 (22.4)

a Three patients had concomitant intoxication via 2 different routes of exposure, resulting in a total of 279 documented exposure routes.

**Table 3.** Poisoning agents.

	<1 year, n (%)	1-5 years, n (%)	6-10 years, n (%)	>10 years, n (%)
Chemical substances				
Pesticides (n=14)	0 (0)	10 (71.4)	3 (21.4)	1 (7.2)
Rodenticides (n=23)	1 (4.3)	20 (87.1)	1 (4.3)	1 (4.3)
Toxic gases or fumes (n=1)	0 (0)	0 (0)	0 (0)	1 (100)
Cleaning agents (n=11)	1 (9.1)	9 (81.8)	0 (0)	1 (9.1)
Petroleum products (n=14)	0 (0)	11 (78.6)	2 (14.3)	1 (7.1)
Other^a^ (n=35)	4 (11.4)	23 (65.7)	6 (17.1)	2 (5.8)
Venom^b^				
Snakebite (n=10)	0 (0)	3 (30)	4 (40)	3 (30)
Insect stings or bites (n=39)	1 (2.6)	15 (38.5)	15 (38.5)	8 (20.4)
Food^c^ (n=58)	3 (5.2)	26 (44.8)	18 (31)	11 (19)
Medications				
Sedatives or sleeping pills (n=9)	0 (0)	4 (44.4)	1 (11.2)	4 (44.4)
Pain relievers (n=15)	1 (6.7)	3 (20)	3 (20)	8 (53.3)
Other^d^ (n=45)	13 (28.9)	27 (60)	3 (6.7)	2 (4.4)
Addictive substances				
Alcohol (n=1)	0 (0)	0 (0)	0 (0)	1 (100)
Illicit drugs or heroin^e^ (n=1)	1 (100)	0 (0)	0 (0)	0 (0)

a Other chemical substances included agents outside the prespecified chemical categories, such as mothballs, cajuput oil, 90% ethanol, petroleum jelly, gel ice packs, and mercury.

b Snakebite refers to snake envenomation, whereas insect stings or bites refers to bee stings.

c Food included poisoning associated with food products, including spoiled or unhygienically prepared food, poisonous mushrooms, and food products or additives of unknown origin.

d Other medications included medications outside the prespecified medication groups, such as antiepileptic drugs, oral contraceptives, vitamin supplements, and traditional medicines.

e Patient was exposed to heroin through breast milk.

Treatment was mostly supportive. Intravenous fluids were used in 80.4% (n=222) of the 276 cases, symptomatic treatment in 35.5% (n=98), gastric lavage in 26.1% (n=72), and single-dose activated charcoal in 24.3% (n=67). More intensive or specific interventions were uncommon, with antidotes used in 6.2% (n=17) of cases and mechanical ventilation used in only 0.4% (n=1) of cases. Outcomes were favorable: 97.5% (n=269) of patients were discharged, 2.5% (n=7) were transferred to a national hospital, and there were no in-hospital deaths (Table 4). A 3-day-old term male neonate developed somnolence, cyanosis, bradypnea, and respiratory failure after documented maternal heroin use while breastfeeding. The treating team classified this as enteral heroin exposure, and the infant was transferred after 3 days. The final outcome after transfer was unavailable.

Although 220 patients presented with poisoning via the gastrointestinal route, only 157 received gastrointestinal decontamination measures (induced emesis, gastric lavage, or activated charcoal) because some patients in this group presented with symptoms such as vomiting, diarrhea, and abdominal pain and were managed with symptomatic treatment and fluid and electrolyte replacement alone; alternatively, some may have already undergone appropriate decontamination before admission and therefore did not meet the indications for this intervention.

**Table 4.** Treatment and clinical outcomes.

	<1 year, n (%)	1-5 years, n (%)	6-10 years, n (%)	>10 years, n (%)
Prevention of gastrointestinal absorption				
Induced vomiting (n=1)	0 (0)	0 (0)	1 (100)	0 (0)
Gastric lavage (n=72)	1 (1.4)	57 (79.2)	7 (9.7)	7 (9.7)
Single-dose activated charcoal (n=67)	2 (3)	48 (71.6)	10 (14.9)	7 (10.4)
Multiple-dose activated charcoal (n=5)	0 (0)	2 (40)	1 (20)	2 (40)
Cathartic (sorbitol; n=12)	0 (0)	7 (58.3)	4 (33.3)	1 (8.3)
Supportive treatment				
Intravenous fluids (n=222)	17 (7.6)	119 (53.6)	47 (21.2)	39 (17.6)
Parenteral nutrition (n=2)	1 (50)	1 (50)	0 (0)	0 (0)
Oral rehydration (n=10)	3 (30)	3 (30)	3 (30)	1 (10)
Mechanical ventilation (n=1)	1 (100)	0 (0)	0 (0)	0 (0)
Symptomatic treatment (n=98)	7 (7.1)	52 (53.1)	26 (26.5)	13 (13.3)
Specific antidotal treatment				
Forced diuresis (n=2)	0 (0)	1 (50)	0 (0)	1 (50)
Hemodialysis (n=0)	0 (0)	0 (0)	0 (0)	0 (0)
Administration of antidotes (n=17)	3 (17.6)	3 (17.6)	4 (23.5)	7 (41.2)
Antivenom serum (n=0)	0 (0)	0 (0)	0 (0)	0 (0)
Outcomes				
Discharge (n=269)	21 (7.8)	149 (55.4)	56 (20.8)	43 (16)
Transfer to a national hospital (n=7)	4 (57.1)	2 (28.6)	0 (0)	1 (14.3)
Discharge against medical advice (n=0)	0 (0)	0 (0)	0 (0)	0 (0)
In-hospital death (n=0)	0 (0)	0 (0)	0 (0)	0 (0)

## Discussion

### Principal Findings

This study showed that acute poisoning was concentrated among children aged 1 to 5 years and most often occurred unintentionally at home. Ingestion was the main route of exposure. The agent profile was mixed, with chemical substances, medications, food, and venom-related exposures all represented. Treatment data are descriptive and should not be interpreted as evidence that specific procedures were appropriate or effective. Most children were discharged from the study hospital, while 7 were transferred to another hospital, and their final outcomes were unavailable.

The age distribution is consistent with recent evidence from Haiphong Children’s Hospital in Vietnam, where children aged 1 to 5 years also represented the largest group (65.6%) and most poisoning cases were unintentional (82.1%) [16]. However, the agent profile differed. In Haiphong, nonpharmaceutical chemicals (42.9%) and medications (37.6%) predominated, whereas the present dataset showed a more mixed pattern that also included substantial food and venom-related exposures. These differences suggest that pediatric poisoning profiles can vary between provincial settings and support locally tailored prevention rather than assuming that one national pattern applies uniformly.

The local agent pattern is therefore an important contribution of this study. Pesticides and rodenticides were present but did not dominate the recorded cases, while food-related poisoning and bee stings were comparatively prominent. This extends the available evidence from Vietnam by providing recent data from Thai Binh and by showing that household, food-related, environmental, medication, and envenomation exposures all contribute to the clinical workload. For local prevention, practical priorities include safer storage of medicines and household or agricultural chemicals, caregiver education for families with young children, and locally relevant messaging about food safety and venomous or stinging animals.

The age distribution of intentional poisoning is clinically important. In this cohort, all intentional poisonings occurred in children older than 10 years. This is consistent with the wider literature showing that intentional self-poisoning and substance-related toxic exposures are concentrated in adolescents, with psychosocial and mental health factors playing a substantial role [4,5,17,18]. This pattern indicates that poisoning prevention cannot rely on a single strategy across all age groups. For younger children, prevention should focus on supervision and safe storage. For older children and adolescents, screening for emotional distress, self-harm risk, and intentional ingestion becomes much more relevant.

The in-hospital outcome data require cautious interpretation. Most children were discharged from Thai Binh Pediatric Hospital, and there were no deaths at the study hospital. However, 7 children were transferred to a national hospital, and their subsequent outcomes were unavailable, so the cohort cannot be described as having uniformly favorable final outcomes. In addition, the retrospective records did not contain enough standardized information on poisoning severity or on the timing, indication, and complications of procedures such as gastric lavage, induced vomiting, activated charcoal, or cathartics to assess treatment appropriateness. The frequencies reported here should therefore be interpreted as patterns of recorded practice rather than evidence of successful management.

These findings have several practical implications. For young children, prevention should prioritize storing medicines, rodenticides, pesticides, cleaning products, and petroleum products in locked or otherwise inaccessible locations. Toxic products should also not be transferred into food or drink containers. The mixed agent profile also supports caregiver education on food safety and rapid recognition of snakebite or bee sting toxicity. For older children and adolescents, the concentration of intentional poisoning in those older than 10 years supports routine assessment of self-harm risk and psychosocial distress when intentional exposure is suspected. At the service level, standardized documentation of exposure intent, agent, timing, severity, treatment indication, and transfer outcome would improve both clinical continuity and future surveillance.

This study should be interpreted in light of several limitations. First, the retrospective design depended on the accuracy and completeness of medical records. Therefore, some variables may have been missing or misclassified. Second, this was a single-center hospital-based study; therefore, the findings may not fully represent the broader community burden of pediatric poisoning or other regions with different exposure patterns. Third, the study was descriptive and could not identify causal risk factors or predictors of poor outcome. Fourth, poisoning severity scores, complete toxicological confirmation, or detailed long-term follow-up were unavailable, which limits the interpretation of treatment appropriateness and the full clinical impact of poisoning. Finally, because there were no in-hospital deaths and relatively few severe outcomes, the study has limited ability to identify factors associated with mortality or critical illness.

### Conclusions

Pediatric acute poisoning at this provincial hospital predominantly affected young children and usually occurred unintentionally in the home. The findings confirm the importance of early-childhood household exposures, as reported previously in Vietnam, while also showing a mixed local agent profile that includes chemical, medication, food-related, and venom-related exposures. Local prevention should emphasize secure storage, caregiver education, food and environmental safety, and age-appropriate assessment of intentional poisoning in older children. Most children were discharged from the study hospital, but final outcomes were unavailable for those who were transferred. Treatment frequencies should be interpreted descriptively rather than as evidence of treatment appropriateness or effectiveness.
